# 800. Needle Stick And Sharps Injuries Among Health Care Workers – The Experience of a Tertiary Hospital in Jamaica

**DOI:** 10.1093/ofid/ofab466.996

**Published:** 2021-12-04

**Authors:** Sue-Ann Martin, Tamara Thompson, Trevor Ferguson

**Affiliations:** 1 University of The West Indies, Mona, Jamaica, Kingston; 7 Kingston, Jamaica

## Abstract

**Background:**

Despite advances in occupational safety protocols in healthcare facilities, needle stick and sharps injuries (NSSIs) continue to be a concerning occurrence among healthcare workers (HCWs). Such exposures lead to the risk of blood borne infections as well as emotional and financial consequences which may be difficult to measure. Few studies on NSSIs among Jamaican HCWs have been published in the last decade. We evaluated adherence to established NSSIs management protocols and investigated the demographic and work-related characteristics of HCWs sustaining NSSIs. The proportion of HCWs affected and frequency and direct costs associated with these injuries were estimated.

**Methods:**

This retrospective case-series reviewed HCWs employed or contracted to an urban tertiary teaching hospital in Jamaica, who reported NSSIs in the emergency room during the period January 1, 2015 and December 31, 2018. Archived incident reporting forms for these HCWs were reviewed. Data were analyzed using STATA 14 statistical software.

**Results:**

57 cases of NSSIs were reported at an average rate of 1 per month and an annual incidence of 0.14 per 100 beds and 4.75 per 100 workers. 49 (86%) HCWs were female and 18 (14%) were male. 55% of HCWs were between the ages of 24-29 years old. Nurses (53%) and physicians (19%) made up most of the sample. 28 (58%) HCWs reported not wearing gloves during the incident. Improperly handling used sharps and re-capping needles were reported by 26 (46.5%) and 17 (30%) of HCWs respectively. HIV and viral Hepatitis screening of source patients were performed 85% and 55% of the time respectively. Only 25% of HCWs were prescribed HIV post exposure prophylaxis. Post-injury counselling occurred with 52 (91%) HCWs and follow up care arranged for 36% of cases. The mean direct cost associated with the initial management of the HCW was US &163.22 per NSSI.

**Conclusion:**

The frequency of reported NSSIs by HCWs is low but the burden of direct costs is high. There is inadequate adherence to NSSIs management protocols and accurate monitoring systems are lacking. We recommend the need for safer needle disposal methods and devices, routine auditing of sharps practices and training in occupational hazard prevention and management. This may improve occupational risk perception among HCWs and workplace safety.

**Disclosures:**

**All Authors**: No reported disclosures

